# GUD-VE visualization tool for physicochemical properties of proteins

**DOI:** 10.1016/j.mex.2023.102226

**Published:** 2023-05-26

**Authors:** Ritu Chauhan, Juhi Bhattacharya, Rubi Solanki, Farhan Jalees Ahmad, Bhavya Alankar, Harleen Kaur

**Affiliations:** a Amity University, Noida 201313, Uttar Pradesh, India; b School of Interdisciplinary Sciences and Technology, Jamia Hamdard, New Delhi 110062, India; c Department of Computer Science and Engineering, School of Engineering Sciences and Technology, Jamia Hamdard, New Delhi 110062, India

**Keywords:** Amino acids, Graphical user interface, Molecular weight, Grand average of hydropathicity, Isoelectric point, GUD-VE Visualization Tool for Physicochemical Properties of Proteins

## Abstract

The physicochemical properties of primary sequences of proteins helps in determining both the structure and biological functions. The sequence analysis of the proteins and nucleic acids is most fundamental element of bioinformatics. Without these elements, it is impossible to gain insight deeper molecular and biochemical mechanisms. For this purpose, the computational methods like bioinformatics tools assist experts and novices alike in resolving issues relating to protein analysis. Similarly, this proposed work, for the graphical user interface (GUI) based prediction and visualization through the computations-based method done on Jupyter Notebook with tkinter package which allows the creation of a program on a local host platform and accessed by the programmer.

• When it is queried with a protein sequence, it predicts physicochemical parameters of the peptides.
• Users can choose to visualize the findings acquired either anonymously or on the user-specified email address and compare the biophysical properties of one protein with other using amino acids (AA) sequences.

The aim of this paper is to meet the requirements of experimentalists, not just hardcore bioinformaticians related to biophysical properties prediction and comparison with other proteins. The code for it has been uploaded on GitHub (an online repository of codes) in private mode.

## Graphical abstract

**Figure:**
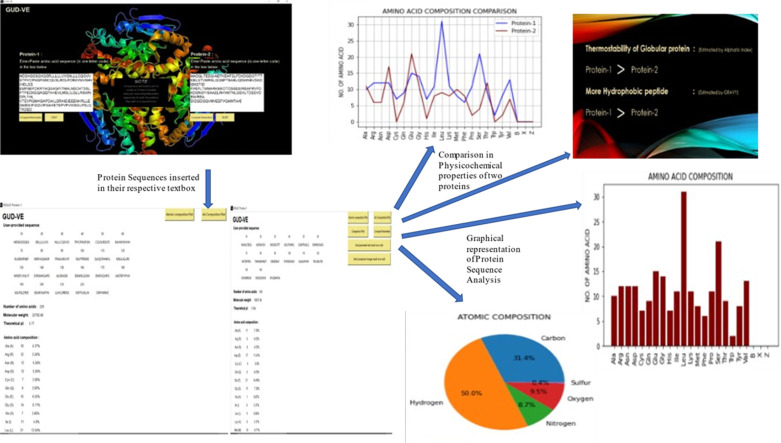

Specifications table

**Table:** 

Subject area:	Bioinformatics
More specific subject area:	Visualization tool application in protein differentiation on the basis of factors
Name of your method:	GUD-VE Visualization Tool for Physicochemical Properties of Proteins
Name and reference of original method:	1.) Gasteiger, E., Hoogland, C., Gattiker, A., Duvaud, S., Wilkins, M.R., Appel, R.D., Bairoch, A., 2005. Protein Identification and Analysis Tools on the ExPASy Server, in: Walker, J.M. (Ed.), The Proteomics Protocols Handbook. Humana Press, Totowa, NJ, pp. 571–607. https://doi.org/10.1385/1-59259-890-0:571 2.) Ikai, A., 1980. Thermostability and aliphatic index of globular proteins. J Biochem 88, 1895–1898. PMID: 7462208 https://www.jstage.jst.go.jp/article/biochemistry1922/88/6/88_6_1895/_article/-char/en 3.) Kyte, J., Doolittle, R.F., 1982. A simple method for displaying the hydropathic character of a protein. Journal of Molecular Biology 157, 105–132. https://doi.org/10.1016/0022-2836(82)90515-0
Resource availability:	*N.A. (it will be provided after the paper is accepted)*

## Introduction

The functions of a protein is defined and given by their unique structure provided by their amino acid (AA) compositions and folds. Proteins with similar AA sequences exhibit similar bioactivity, similar structure, and function. However, Point Mutations, deletions, or alterations in the expression of the genes results in mutant proteins leading to many incurable diseases; proteins translated by these genes have different AA sequence than native protein which leads to the variation in the structure of proteins, different structures lead to different functions or deregulation in the biological processes. These deregulated biomolecules, such as nucleic acids and proteins, more specifically enzymes, cytokines, antibodies, aptamers, etc. which are crucial for certain complex diseases pathogenesis such as cancer, diabetes, Alzheimer's, Parkinson's disease, are known as biomarkers. The identification of biomarkers that are indicative of a specific biological state, is a major research topic in biomedical applications of computational biology [1], [2], [3]. Thus, the protein's family, superfamily, subcellular localization, physicochemical properties give insight to the researchers for further research like peptide binding sites prediction, drug development, functional annotation, disordered protein etc.

Proteins are depicted as having diverse roles in the body because of their functions such as catalysts, cofactors, hormones, transporters, structure, signaling for biochemical reactions, maintaining physiology and many more. Therefore, it becomes necessary to examine the protein sequence and structure [2]. To study the protein structure in vivo and in vitro requires technical expertise, resources, and financial implications. Earlier many innovative and user-friendly research tools and software were developed to make it easier and cut down these complications and diminish the line between experimental result and information technology based analysis [4], [5], [6]. The First protein sequence analysis tool was reported in the year 1965, named COMPROTEIN. The Dayhoff and Robert S. Ledley'Atlas' first version included 65 protein sequences, the majority of which were interspecific variations of a few proteins. As a result, the first Atlas was a perfect data set for two researchers who theorized that protein sequences indicate a species' evolutionary history. Because of the massive increase in the number of protein sequences and structures recorded in biological databases, several bioinformatics tools, and systems have been developed to organize, validate, compare, and analyze this massive amount of data. It is now maintained as the PIR- International [7,8]. Furthermore, effective algorithms to utilize this sequence and structural data to aid protein classification and infer the biological function of newly found proteins are being created.

A catalog of bioinformatics resources known as ExPASy SIB Bioinformatics Resource Portal has several protein sequence analysis tools for biophysical properties among other key resources. Online tools Compute pI/mW, ProtParam, ProtScale, and AAcompIdent are related to the functionalities of our tool which are discussed here [9]. Compute pI/mW (isoelectric point/ molecular weight) tool is available at ExPASy which predicts the isoelectric point and molecular weight of proteins. It takes input as UniProtKB/ Swiss-Pot Protein ID/ UniProt Knowledgebase accession number as input. It can predict the pI/mW for many proteins at once [10]. The ExPASy ProtParam calculates physicochemical parameters of the protein. This tool Computes both pI and mW as well as predicts AA composition, atomic composition, extinction coefficient, estimated ½ life, and instability index among others. It takes input in the form of an AA sequence of the protein or Swiss-Prot/TrEMBL accession no. or ID [10]. ProtScale is used to compute and display the protein profile using 57 amino acid scales, such as hydrophobicity, hydrophilicity charts. These tools require just amino acid sequence to give the output, no additional information is required [10]. AAcompIdent compares the theoretical percent of AA composition in the Swiss-Prot/ TrEMBL database to the empirically measured percent of AA composition of an unknown protein. The extent of the difference in the composition of unknown protein and protein entry in the database is calculated by a score. This score is the sum of squared between the percent AA of the all AA in unknown protein and the protein in the database. Then the matched proteins are ranked in the list with respect to this score. The best matches are preserved at the top of the list, while the poorest are retained at the bottom [10]. PepDraw allows users to draw chemical structure of AA sequence entered in the input box given on user interface along-with peptide analysis (http://www.tulane.edu/~biochem/WW/PepDraw/). Most of these tools give individually computed parameters. No option is available to compare the biophysical properties computed for a query protein sequence with other protein in these above-mentioned tools. Lack of interactive interface is also there while using these tools. Instances of microheterogeneity can be identied by using our program. In classical biochemistry methods, solutions of high concentrations are required to calculate the physiochemical properties of proteins which our program predicts and compares with other proteins AA sequence.

Hence, our proposed work has been created to investigate protein's physicochemical properties. This program provides precise results and a user-friendly interface. It compares the two protein sequences, using a set of parameters such as atomic composition, molecular weight, theoretical pI, AA composition, number of amino acids present in our AA sequence, aliphatic index, and GRAVY. These parameters can be used in various studies like protein structure prediction, function prediction, mutation prediction, etc. It also generates output in the form of graphs and pie charts, such as amino acid composition and atomic composition. The sequence of AA from the protein database can be used to investigate protein's properties. This application is made up of three parts: a graphical user interface, data visualization, and e-mail. Python's tkinter module is used to create the user interface. There are three windows in all. The primary window is where the user enters the target sequences of proteins that need to be compared. The parameters are displayed in other sub-windows. We used visual data approaches for improved visualization so that the user can easily understand the parameter analysis. Protein thermostability and hydrophobicity are also compared by GUD-VE. The aliphatic index and the grand average of hydropathicity (GRAVY) are used to compute this. It not only provides you with the output but also allows you to preserve it for future use by providing you with the option of receiving and saving your output by email. This will aid in the identification of proteins based on their AA compositions.

## Method details

The GUD-VE architecture not only computes the parameters individually but also compares the user-provided sequences using data visualizations like graphs and comparison templates. This proposed work is done on the Jupyter Notebook using tkinter package on the local host, accessed by the programmer only.

### Architecture design

**Fig. 1:**
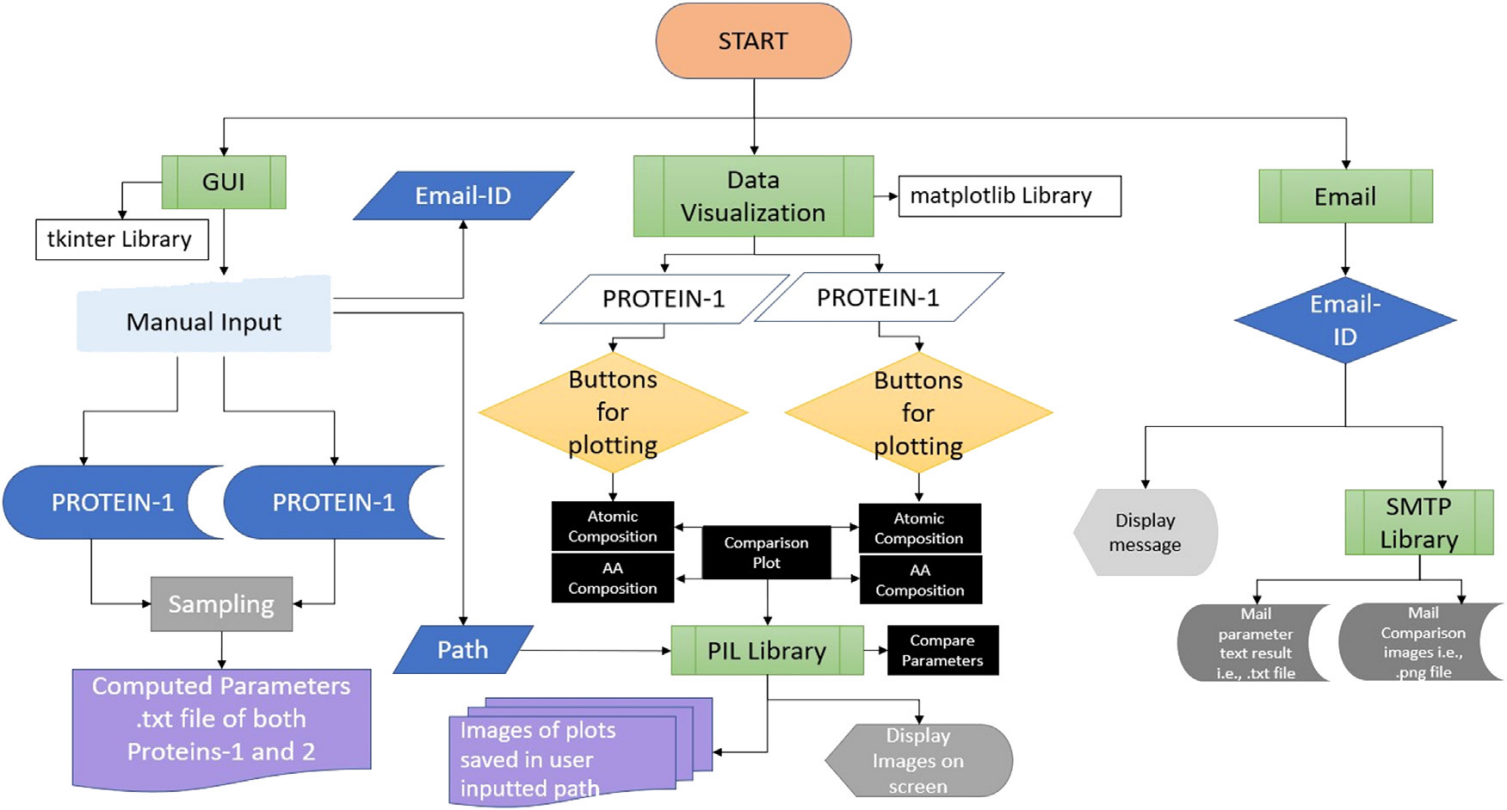
GUD-VE's pipeline showing the feature selection processes are completely parallelizable and when all the parameters are fulfilled completely the feature modules are enumerated and result computation starts for Protein-1 and Protein-2, individually. Feature selection methods include Graphical User Interface (GUI), Data Visualization.

This proposed work's architecture is based on three domains: Graphic User Interface (GUI), Data Visualization, and E-mail. The GUI of the application is developed on the tkinter Library. The Data Visualization is developed on the matplotlib Library of Jupyter Notebook. The e-mail is developed on smtp Library of Jupyter Notebook. The flowchart is depicted in Fig. 1.

### Architecture principle

Its architecture is divided into three parts namely GUI, Data visualization, and Email. The first root window of this proposed paper work, was made using the tkinter library is designed to take user input of two protein sequences, an Email address if reports to be mailed and a user-desired path to save the plots. Buttons labeled ‘submit’, ‘reset’, ‘compute parameters’ are also there, to perform their namely functions. This proposed work allows a user to calculate the parameters for a single protein if required, keeping this in mind it is designed to open computed results in two different root windows. These input of protein sequences are used for calculating the parameters, which are elaborated in later parts of this work and used for making plots and charts for data visualization. PyPlot from Matplotlib library is used for this part along with PIL library for saving and opening created plots and charts into and from the memory location.

Users can also receive the report of computed parameters in form of a text document and plots in a png format attached and sent to them via email, stmplib library is used for this, which uses stmp.google.com and its port for sending emails. So, the architecture is named GUD-VE for GUI, Data Visualization, and Email, which works on user inputs of protein sequences, recipient email address, and a memory directory path.

### Architecture prototype

This application uses data visualization and a graphical user interface to make the platform of the application more user-friendly. In this section, we describe the procedures of the program separated into three main components which are graphical user interface, data visualization, and email.

#### Graphical user interface (GUI)

The programming of application is based on Python programming language. The GUI framework of the application is done using the tkinter library of python. Tkinter is the standard GUI library for Python. It provides a powerful object-oriented interface to the Tk GUI toolkit. The programming of this application is done such that there are three operational root windows. The first is the main window and the remaining two windows contain the parameters of the protein sequence provided by the user namely, Protein-1 and Protein-2. This could be achieved by importing various widgets from the tkinter library, such as Labels are used to place the required text on the window. The Button widget is used to give the command to the application to perform a particular function. Entry widget used to take information from the user like email ID and path. Text Box widget used to incorporate the protein sequences whose parameters need to be determined and compared. The Algorithm 1 for GUI, we utilized to develop this work is given below.

**Algorithm 1:** For GUI of Protein-1 and Protein-2.

1: Import the tkinter library
2: Create and place the Label ‘Protein-1′ on the canvas of the first root window
3: Create and place Label that informs the user to give manual input beneath the previous label
4: Create and place a button that contains command to ‘Compute Parameters’ below the previous label
5: Create and place a button that contains command to ‘RESET’ below the previous label
6: Create and place a text box where the user will give the manual input

##### Repeat the above steps for ‘Protein-2′ main root window

**Fig. 2:**
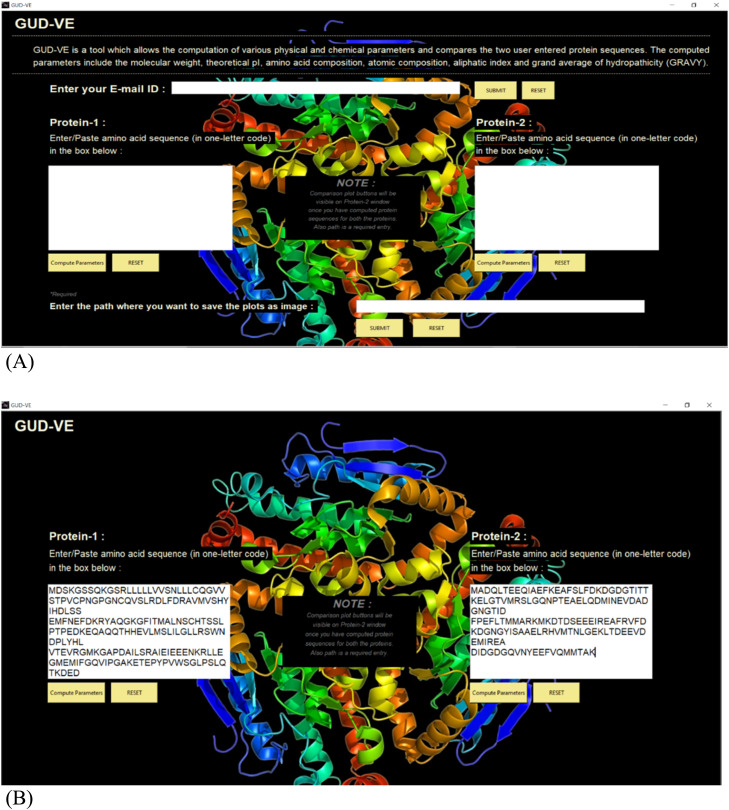
GUD-VE homepage before submission is given in (A) and after submission is given in (B) of the query protein sequences.

Labels are used to display the text that the user needs to get informed about regarding the working of the application. There were two-button widgets that were used namely, “COMPUTE PARAMETER” and “RESET” buttons. These buttons take command from the user when they have entered the text or provided the apt information as per the requirement of the application for it to compute the parameters or if the user entered the wrong protein sequence it can be reset to its original empty text box form, respectively. The entry widgets and the text box widgets are used in the program to take input from the user in a single line and multi-line format, respectively. In this application, these widgets have been used to take the input like the email address, path, and the two protein sequences from the user that needs to be computed. Fig. 2. is given below to show the homepage of this proposed work for both before submission of AA sequence given in Fig. 2(A) and while submitting the AA sequence is given in Fig. 2(B)

This application takes the amino acid sequences of two proteins labeled as Protein-1 and Protein-2. For protein analysis, information in protein databases can be used to predict certain properties about a protein, which can be useful for its empirical investigation. This is what happens when the user presses the compute parameters button. Our proposed research work makes it simpler and thus takes two protein sequences instead of one and automatically compares their parameters along with the help of graphs. Thus, reducing the manual workload of identifying and comparing the proteins. The layout of the above widgets has been designed on the tkinter canvas and frames. Following are the features of the application and an explanation of its GUI. Text boxes have been placed beneath the labels of Protein-1 and Protein-2.

The next feature in this application is that it takes the path as input from the user i.e., the location where the user wants to save the images of the plots that they want to save on their personal computer. This has been obtained using the PIL Python Image Library. The images will be saved in .png format in the assigned location with the name ‘c’ and ‘p’ for comparison template and plots, respectively. The input for the path is a required entity.

The next and the most salient feature of this application is that the user can send the protein parameters and compared results obtained for both the proteins via e-mail. The GUI design for this is the entry widget that takes the email ID of the user as input and the “SUBMIT” button which helps to store the email address in the program. The above-mentioned E-mail feature in the application has been implemented using python library email.

##### Sub-root windows

**Fig. 3:**
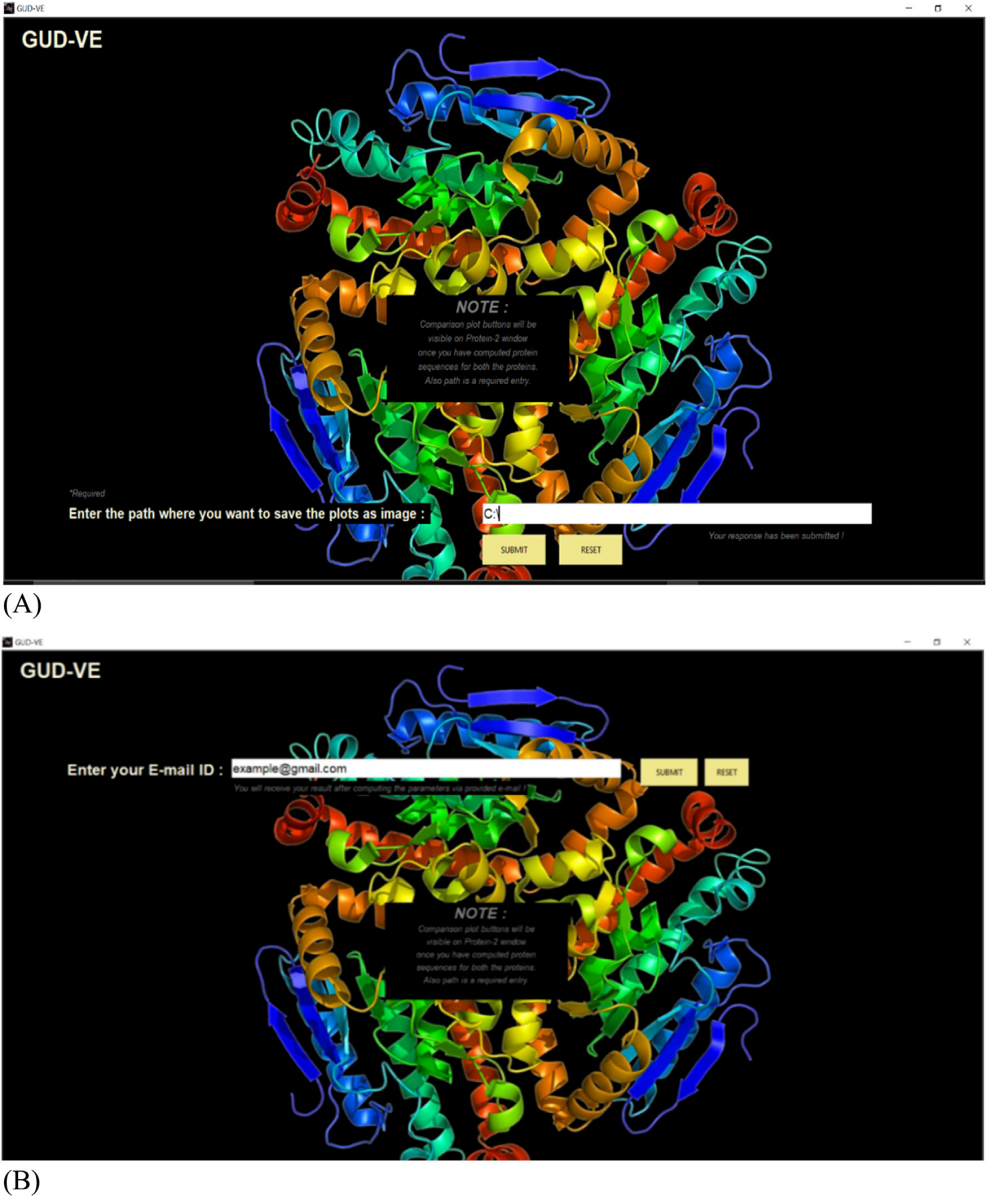

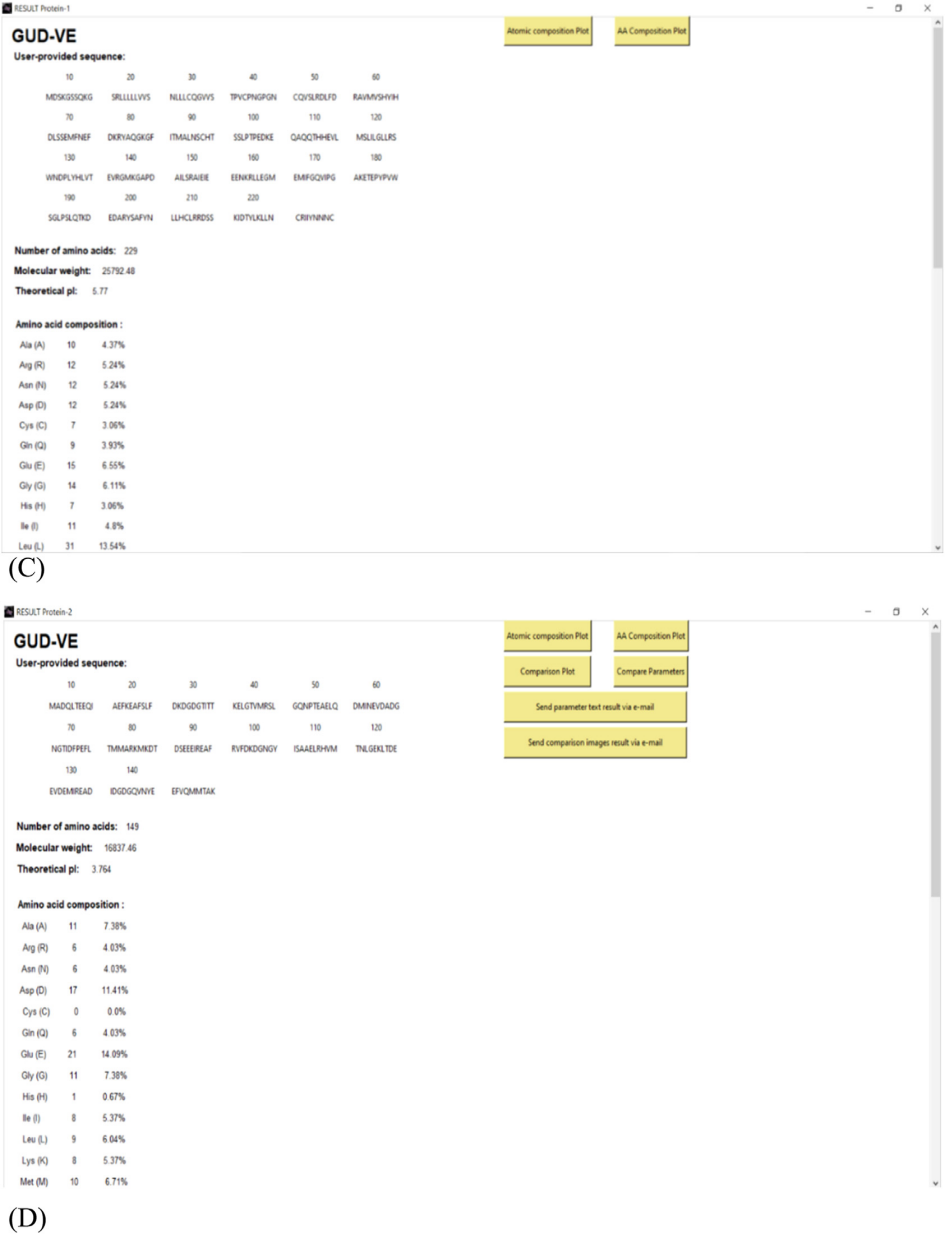
**(A), (B)** Screenshot of this proposed work to show how the path can be given to save the output on the system and on the given E-mail address user provides. Fig. 3**(C), (D).** The sub root window sample screens for Protein 1 and Protein 2 have been shown here in (A) and (B) respectively showing the user provided protein sequence; number of amino acids, molecular weight, theoretical pI, and amino acid composition calculated.

The outset of the sub root windows for Protein-1 and Protein-2 is connected to the “COMPUTE PARAMETER” button attached to the main root window. In Fig. 3(A) and (B), it is shown the results can be downloaded on the location through the path user provide or on the E-mail address on the basis of user's choice. Users can save results on both the local address and E-mail address. The parameterized data based on the molecular weight of amino acid, number of amino acids, theoretical pI, amino acid composition, atomic composition, etc. have been displayed on this window using labels as shown in the Fig. 3(C) and (D) given below.

The buttons for further data visualization have been anchored to the top right corner of the windows. These buttons are labeled as Atomic Composition Plot and the AA Composition Plot. These buttons have the command that is associated with the plotting of graphs using matplotlib library which has been explained later in this report. The first Protein-1 window contains two buttons as mentioned before but the Protein-2 window contains maximum of six buttons including the comparison plot, the compare parameter template, and the rest two buttons are associated with sending mail to the user-mentioned email ID. These buttons are labeled as “Send parameter test result via e-mail” and “Send comparison images result via e-mail” which compiles the protein parameters of both the proteins into one .txt file and which attaches the comparison plots and template in .png format in the body of the email and sent to the user mentioned email address separately, respectively. Thus, providing the users the choice to enter the email ID or path.

The exceptional case in which these buttons won't be visible is when the only parameter for Protein-2 has been computed and also the comparison plot buttons won't be visible since there is no Protein-1 to compare it to. Another exceptional case is if the amino acids B (Aspartic Acid or Asparagine), X (unspecified amino acid), or/and Z (Glutamic acid or glutamine), are present in the protein sequence. These amino acids are ambiguous, and their atomic acid composition cannot be estimated. So, the button for the atomic composition plot is not visible.

#### Data visualization

In this proposed work, along with data output of computed parameters, visual data representation techniques are used for a better understanding and analysis of those parameters it computes. Matplotlib library of python programming language is used for this purpose. This application calculates various physicochemical properties of AA sequence provided in the first root window, is used to make plots and charts. Our proposed work takes two protein sequence inputs and then assesses them to plot two types of graphs and one pie chart. First is a Bar-plot which shows the percentage composition of amino acids. The pie-chart is used to visualize the atomic composition of that protein and an overlapping line-plot is used for a head-to-head comparison of amino acid composition. Additionally, this proposed work also compares aliphatic indexes and hydrophobic characters of two proteins which helps to determine more thermostable and hydrophobic characters having protein. These two parameters are also expressed in visual form on an image template that can be viewed and saved to a user-defined location.

While **plot1()** function is used for making a bar graph, **plot2()** is used for making a pie-chart of the atomic composition of that protein whose sequence the user provides. Like function **plot1(),** function **plot2()** also takes one argument of list datatype with computed atomic composition from another block of code and then makes the pie-chart.

From matplotlib library, **plt.pie()** function is used, it takes two arguments, first, one is of computed atomic composition and second for labels on pie-chart, but instead of a list, manual names are entered inside **plt.pie()** because only five atoms are computed, the third argument is **autopct='%0.1f%%'** which shows the percentage on pie-chart sets the output text up to 1 decimal point. **Plt.title()** is used to set the title of this pie-chart as “ATOMIC COMPOSITION”.

**Image.save()** and **Image.open()** are used to save this pie-chart in .png format and to access and open that saved pie-chart respectively. And this pie-chart is also opened up in a new window for both the proteins separately.

The bar-graph and pie-chart made for each protein are handy to evaluate one protein's parameters but to visualize their difference in AA composition an overlapping line-plot is used in this proposed work for base-to-base comparison.

Function **plotcom()** is defined for making this overlapping line-plot, and it takes two variables as arguments, these two variables are the amino acid composition of the first protein and second protein, these two parameters are required to make a comparative plot, rest part of this function uses the same methodology as of other functions, again matplotlib library is used, **plt.plot()** function is used separately for each protein, it overlaps the line plot of second protein on the first one.

Apart from amino acid composition and atomic composition, this proposed work also calculates thermostability of globular protein using aliphatic index and predicts more hydrophobic protein using the Grand Average of Hydropathicity (GRAVY) method.

To show which protein is more thermostable and more hydrophobic, this proposed work has one saved template, which is used to edit and is saved with the help of ImageDraw library of Python. Function **com()** takes 4 arguments, which are aliphatic indexes of the two proteins and GRAVY values for both. It simply compares those two parameters of two proteins and then draws either a ‘ **>** ‘ or a ‘ **<** ’ on the template for two parameters.

#### E-mail

In the proposed work the parameters are calculated via the FASTA sequence provided is visible on the output root window, but for future reference or user ease these computed parameters can be mailed via email to a user-provided email address. A report will be attached in a .TXT format file having parameters for both the proteins computed. Function **send()** is defined for this purpose, having the email address and password of sender's email, and takes recipients email address from **email.get()** function. **Stmplib** library was used for this, it is easy to use and flexible library for handling the mail- related tasks.

The text file that has computed parameters is attached via **set_payload()** and encoded with **encoders.encode_base64()**. This encoded text file is attached and sent via stmp of the gmail i.e., stmp.google.com with port number 587, server checks login credentials and if found correct, text file is sent, and the session is ended with **quit()** at the end of the block.

To send plots by mail, **Stmplib** library is used, same as **send()** function, **send_img()** function have the sender email address and password for authentication, to attach the plots, a path variable **path** is defined which have the location of saved plots and is used to open the png format images in **open()** function in **‘rb’** mode. Then data of these plots are attached using **msg.add_attachment()** which takes image data, type, and subtype as arguments, for image these are defined as ‘image’ and ‘png’ in the subtype.

### Calculations

The parameters included are described along with the calculation base for this proposed work as given below:

User-provided sequence, which displays the amino acid sequence divided into 10 fragments each making it easy for the user to demarcate the position of each amino acid in the sequence. The number of AA displays the total number of AA present in the protein sequence. It is calculated by obtaining the length of the amino acid sequence. Molecular weight, which displays a mass of the protein sequence, based on 12 as the atomic number of carbon-12. It is calculated by summing the average isotopic masses of amino acids in the protein and the average isotopic mass of one water molecule.

The amino acids (in three-letter format) included are: 'Ala', 'Arg', 'Asn', 'Asp', 'Cys', 'Gln', 'Glu', 'Gly', 'His', 'Ile', 'Leu', 'Lys', 'Met', 'Phe', 'Pro', 'Ser', 'Thr', 'Trp', 'Tyr' and 'Val'. Theoretical pI, which is calculated using pK values of AA, which were defined by examining polypeptide migration between pH 4.5 and 7.3 in an immobilized pH gradient gel environment with 9.2 M and 9.8 M urea at 15 °C or 25 °C. Amino acid composition, displays the name of the amino acid under the first column, the occurrence of each amino acid in the sequence under the second column, and its percentage composition in the sequence under the third column [12,13].

A total number of negatively charged residues, which displays the sum of the total number of Aspartic acid and Glutamic acid. The total number of positively charged residues displays the sum of the total number of Arginine and Lysine [14]. Atomic composition, which displays the total number of carbon (C), hydrogen (H), Oxygen (O), Nitrogen (N), and Sulphur (S) present in the sequence. The first column contains the name of the atoms. The second column contains the symbol of the atoms, and the third column contains the number of each atom in the sequence.

Formula, which displays the formula of the amino acid sequence using the number of carbon atoms, hydrogen, oxygen, nitrogen, and sulfur atoms data. The total number of atoms, which displays the total number of atoms in the amino acid sequence. It is calculated by summing up the number of carbon atoms, hydrogen, oxygen, nitrogen, and sulfur atoms present in the sequence.

Aliphatic index, which is calculated and displayed according to the following formula given in Eq. (1).

(1) Aliphaticindex=X(Ala)+a×X(Val)+b×[X(Ile)+X(Leu)]

where X(Ala), X(Val), X(Ile), and X(Leu) are mole percent (100 × mole fraction) of alanine, valine, isoleucine, and leucine. The coefficients a and b are the relative volume of the valine side chain (*a* = 2.9) and Leu/Ile-side chains (*b* = 3.9) to the side chain of alanine. This is one of the positive factors for the thermostability of proteins [15].

GRAVY is calculated by summing the hydropathy values of each amino acid in the protein sequence, divided by the number of residues in the sequence [16].

## Results

This section includes the results obtained from evaluating the data inputted by the user. The result has been demonstrated separately under three headings as mentioned before in the methods section.

### Graphical user interface (GUI)

The graphical orientation has already been explained in methodology GUI section 2.3.1 using the tkinter library. The final framework looks like Fig. 2 including all the three sections for manual input i.e., E-mail ID, Protein-1 and Protein-2 sequences and path. when each feature selection process is approached by the user, First, comes the protein sequences. In this part, the user has been informed to enter the amino acid sequence in the box below. This architecture is built such that it takes input as the protein sequence from the FASTA sequence. The user can either paste the sequence from any website of NCBI or it can be inputted manually as per the user's choice. Second, comes the path where the user wants to save the plots as images. Here the user is required to copy the path from their personal computer where they want to save the images that pop up while execution of the program after pressing the buttons. This becomes a required parameter otherwise data visualization and email are not possible.

Third, comes the e-mail feature where the user must enter the email ID of the receiver i.e., whoever they want to send the result to. The result for the main root window has been demonstrated in the previous context. Now, the outcome of the sub root windows will be elaborated. The sub root windows get activated by pressing the “Compute Parameters” button, which has been provided for each protein i.e., Protein-1 and Protein-2 separately. The sub root windows contain the computed parameters and buttons for obtaining the image results as given in Fig. 3**(A) and (B)**.

### Data visualization

**Fig. 4:**
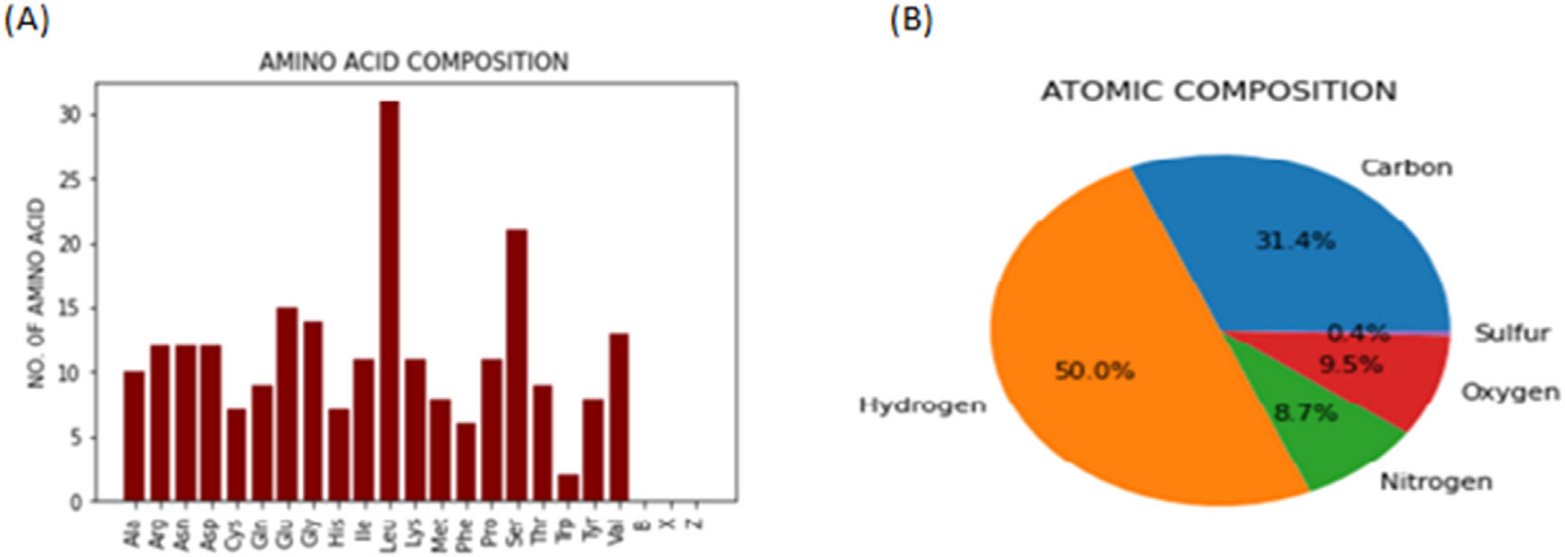
(A), the Bar graph chart is shown representing the number of amino acids in the query protein sequence, and in Fig. 4(B), Pi-Chart is showing the atomic composition for the query protein sequence.

Explained in the previous section the number of amino acids or the amino acid composition is represented in a form of a bar graph for visual representation, three-letter abbreviations are used for amino acid names on the x-axis, and the number of repetitions on the y-axis. This bar graph given in Fig. 4**(A**) can be accessed from the sub-root window separately for both the proteins from their respective windows. It opens in a new pop-up window using the PIL library.

The second available visual representation is a pie-chart showing the atomic composition of the protein with names of the atoms as the label and percentage composition as tags, this pie-chart is also available for both the proteins on both the sub-root windows. Fig. 4**(B)** shows the type of pie-chart that opens when the “atomic composition plot” labelled button is pressed.

**Fig. 5:**
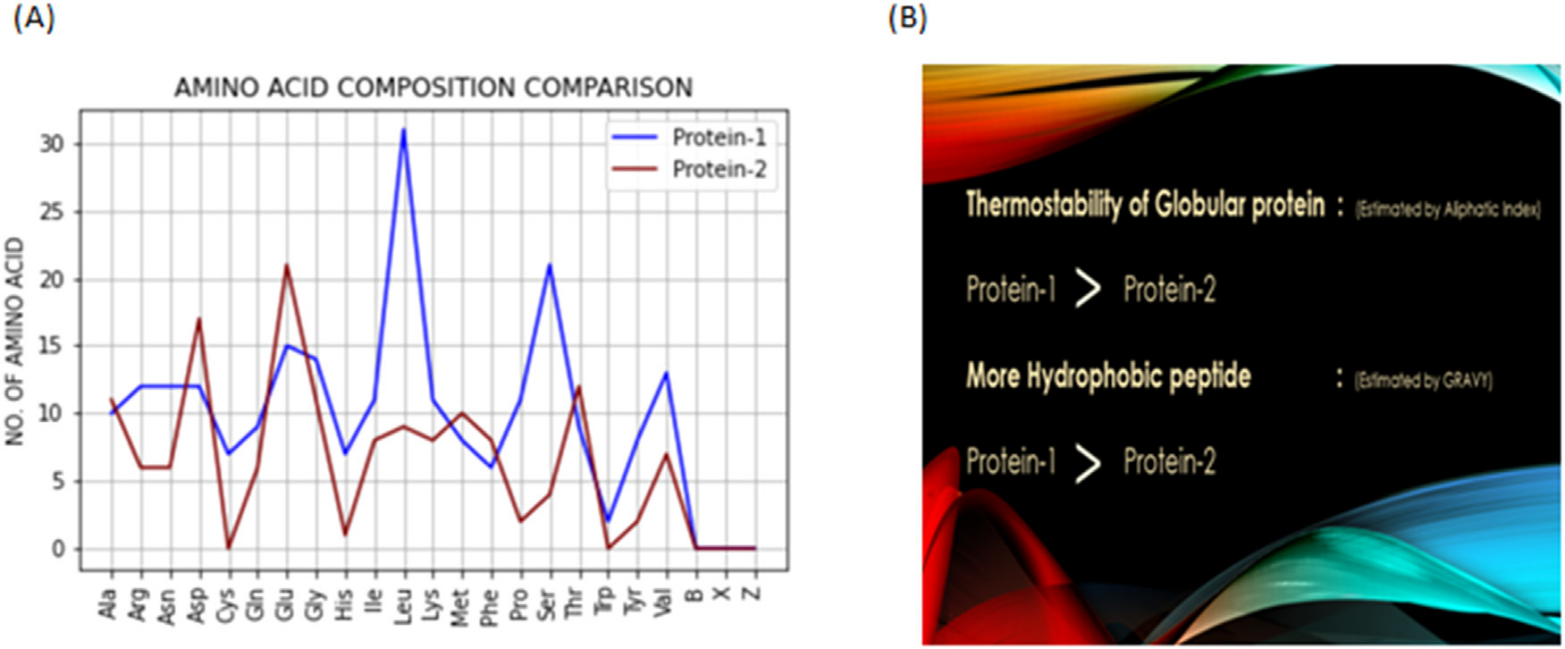
In Section (A), Line graph plot for comparison of AA in two protein sequences obtained through the button labeled as Comparison Plot. In Section (B), the comparison of thermostability and hydrophobicity obtained through prediction is compared in the above-given manner for Protein-1 and Protein-2.

After these two plots for a single protein, for comparison a line graph is plotted which shows one on one overlap for every single amino acid present in both the proteins. This graph is plotted after taking details about both the proteins, this plot Fig. 5**(A)** can be accessed from the second sub-root window through the button labeled as “comparison plot”, which pops a new window with this image.

The last button labelled as “compare parameters” opens a run-time edited image template which compares two important aspects of proteins, the first one being thermostability, calculated using the aliphatic index and hydrophobic nature, calculated using the GRAVY method, both described in the earlier section are compared for both proteins to show which protein is more thermostable and more hydrophobic. Fig. 5**(B)** shows that protein 1 is more thermostable and more hydrophobic as compared to protein 2.

### Saving results for future reference

The parameters and comparisons done for two proteins and the plots generated can be sent to the user if he wants to receive a copy of the report and the plots and seen at the user's end. Computed parameters report is sent in .txt format and comparison report for both the proteins is sent in the form of .png format plots in the attachments to the given E-mail address and both can be downloaded and saved for future reference.

## Discussion

Proteins physicochemical properties have a direct relation with their biological functions, especially GRAVY, thermostability, length of AA sequence, pI, and mW [17]. Proteins belonging to different species can also identified on the basis of the physicochemical properties of the AA sequence of proteins [11]. Furthermore, an advantage of our proposed work is that it utilizes computational methods that can both effectively predict and then compare the physicochemical properties of proteins which may help researchers in reducing cost as well as the time spent in providing a better understanding of the physicochemical properties of Proteins.

Unfortunately, there is a limited study done so far that provides the reasonable prediction and analysis of physicochemical properties of proteins. Cellular assays may be employed for quantifying the mutation's effect on the function and AA change's effect on protein's properties. There is another framework to integrate the protein's sequence data and variation information, its effect on physicochemical properties to interpret the information through bioinformatics-based methods. Combining these two approaches is necessary for the reliability of research and medical implementation [18], [19], [20].

There are many aspects of proteomic research which can be dealt using physicochemical properties of proteins. Identifying the disordered protein regions, peptide binding sites, differentiating the favorable and not favorable AA residues in water soluble and transmembrane protein like bioinformatics research is already done using physicochemical properties of AA sequences. Disordered protein region was identified with the help of biophysical properties of proteins because they mostly have polar and charged AA and their depletion takes place in hydrophobic AA. physicochemical properties of AA sequence which are hydrophobicity, volume, area, pI, and the indicator variables aliphatic, aromatic, branch and sulfur were used to predict the short peptide binding with Major Histocompatibility Complex [21], [22], [23]. Herein, we have proposed work that compares the two protein sequences and provides powerful computation of different physical and chemical parameters.

Furthermore, this proposed work provides the user a text file that includes the number of AA, molecular weight, theoretical pI and AA composition also the information in three types of plot amino acid composition, atomic composition, and comparison between two protein sequences. Thus, allowing us the new interpretation of underlying physicochemical behavior in the query protein sequences. This proposed work's architecture not only computes the parameters individually but also compares the user-provided sequences using data visualizations like graphs and comparison templates. The most salient feature of this application is that it can send the computed data to a user-provided e-mail which makes the application more user-friendly.

The first limitation of this proposed work is that it compares and analyses only two amino acid sequences. Since the calculations of the protein parameters are completely programming-based and the BioPython module is not used, the result of some exceptional protein sequences containing ambiguous amino acid sequences can be sometimes incorrectly predicted. Secondly, if, by mistake the user copies a faulty amino acid sequence, the tool will not show any error message. Moreover this program is not a server-based, thus the outcomes are limited to the data programmed in it. It is not supported by mainstream bioinformatics organizations, so the information is limited. We will further extend this proposed work to overcome these limitations and extend the physicochemical properties this proposed work can compute, compare and visualize.

## Ethics statements

Not Applicable.

## Funding

The present study was supported by the 10.13039/501100001501University Grant Commission (UGC), Ministry of Social Justice, New Delhi, India (Grant No. F1-17.1/2015-16/RGNF-2015-17-SC-HAR-20221).

## CRediT authorship contribution statement

**Ritu Chauhan:** Conceptualization, Formal analysis, Supervision, Project administration, Writing – review & editing. **Juhi Bhattacharya:** Conceptualization, Methodology, Software, Writing – original draft. **Rubi Solanki:** Conceptualization, Methodology, Software, Writing – original draft. **Farhan Jalees Ahmad:** Conceptualization, Formal analysis, Writing – review & editing. **Bhavya Alankar:** Conceptualization, Formal analysis, Writing – review & editing. **Harleen Kaur:** Conceptualization, Formal analysis, Supervision, Project administration, Writing – review & editing.

## Declaration of Competing Interest

The authors declare that they have no known competing financial interests or personal relationships that could have appeared to influence the work reported in this paper.

## Data Availability

Data will be made available on request.
